# Effect of consumption of micronutrient enriched wheat steamed bread on postprandial plasma glucose in healthy and type 2 diabetic subjects

**DOI:** 10.1186/1475-2891-12-64

**Published:** 2013-05-17

**Authors:** Lan Su-Que, Meng Ya-Ning, Li Xing-Pu, Zhang Ye-Lun, Song Guang-Yao, Ma Hui-Juan

**Affiliations:** 1 Institute of Cereal and Oil Crops, Hebei Academy of Agriculture and Forestry Sciences, no. 162 Hengshan Street, Gaoxinqu, Shijiazhuang 050035, P. R. China; 2 Hebei General Hospital, Shijiazhuang, P. R. China; 3 Hebei Research Station of Crop Gene Resource & Germplasm Enhancement, Ministry of Agriculture, Shijiazhuang, P. R. China; 4 Key Laboratory of Genetics and Breeding of Hebei Province, Shijiazhuang, P. R. China

**Keywords:** Diabetes, Glycaemic index, Food choice, Functional foods, Health eating

## Abstract

**Background:**

Steamed wheat bread have previously been shown to induce comparatively high postprandial plasma glucose responses, on the contrary, buckwheat products induced lower postprandial plasma glucose. The present study was to assess the effects of micronutrient enriched bread wheat variety Jizi439 and buckwheat on postprandial plasma glucose in healthy and diabetic subjects comparing with buckwheat and other bread wheat varieties.

**Methods:**

Two experiments were conducted to study the effects of bread wheat variety Jizi439 on the postprandial plasma glucose levels of the randomly selected subjects. The first experiment involved three types of steamed bread with equivalent of 50 g available carbohydrate fed to 10 normal weight young healthy subjects. Two types of steamed bread were made from two purple-grain bread wheat varieties, Jizi439 and Chu20, respectively, and the third type was made from the mixture of different white grain wheat varieties. Plasma glucose levels of each subject were measured at 15, 30, 45, 60, 120 min after eating. Glucose was used as a reference, the total area under curve (AUC) and glycemic index (GI) was calculated for test meal. The second experiment was performed among ten type 2 diabetics who were served equivalent of 50 g available carbohydrate of steamed bread made from Jizi 439, the mixture of white grain bread wheat and buckwheat, respectively. The plasma glucose increment was determined two hours thereafter.

**Results:**

In the first experiment, consumption of the steamed bread made from Jizi439 resulted in the least increase in plasma glucose and the GI was significantly lower than that of Chu20 and the mixture. In the second experiment, the average of postprandial 2 h plasma glucose increment of Jizi439 was 2.46 mmol/L which was significantly lower than that of the mixture of white wheat but was not significantly different from buckwheat.

**Conclusions:**

The results indicated that consumption of Jizi439 steamed bread resulted in significantly lower plasma glucose in both healthy and diabetic subjects, compared with other types of test foods, except buckwheat bread. The steam bread made from Jizi439 would be an ideal food for preventing and treatment of diabetes.

## Background

Type-2 diabetes (Diabetes mellitus) is a common chronic disease caused by a fault in the insulin production in the body. Insulin resistance is an important underlying factor. The disease has inheritable tendency. The World Health Organization reported that diabetes has become a major non-infectious disease threatening human health in the world after malignant tumor, Cardiovascular and cerebrovascular disease [1]. It was estimated that 30 million people suffered from diabetes in the world in 1985. By 2000, the number of global diabetics had reached 171 million. And based on this trend, it may reach 366 million in 2030 [2]. Extensive studies have indicated that uncontrolled diabetes may cause serious complications including retinopathy, nephropathy, and neuropathy [3-5]. However, these complications are preventable through the glycemic control [2,6].

It is well known that diabetes is highly correlated with the life style and diet. Proper diet can control plasma glucose level and help to prevent the development of type 2 diabetes. Postprandial hyperglycemia was increasingly recognized as an independent risk factor for cardiovascular disease [7]. Postprandial glycemia can be reliably controlled by considering both the amount and types of carbohydrate. Carbohydrates consist of sugars (e.g., fructose vs glucose), starches (e.g., amylose vs amylopectin) and fibers (e.g., soluble vs insoluble). Fibers are not converted to glucose, cannot be broken down by digestive enzymes and pass relatively intact into the large intestine.It was reported that the Glycemic Index (GI) of a food is influenced by many different factors including the type of sugar, the type of starch, the type of fiber, the processing of a food, and the presence of fat or protein in a mixed meal [8].

There is a large amount of evidence that consumption of low GI foods could have a significant impact on the amelioration of metabolic disturbances [9-11]. The GI is defined as the incremental area under the plasma glucose response curve of a 50 g available carbohydrates portion of a test food, expressed as a percentage of the same amount of available carbohydrate from a reference food (either white bread or glucose) taken by the same subject [8]. The GI can be used to classify foods based on their plasma glucose raising potential. Bread wheat is one of the most consumed food staples globally. In Northern China, steamed bread is a popular daily food. Some studies have evaluated the glycemic response of different wheat bread and reported that was influenced by cereal fibre, baking process and the bread fermentation [12-14]. Several studies have evaluated the glycemic response of steamed wheat bread, and reported that it was normally with high GI (88.1-98.3) [15,16].

In present study, the objective was to find the best bread wheat variety which can be used to make steamed bread with low GI. A purple-grain wheat variety named Jizi439 developed by ourselves, with riched micronutriton especially organic chromium which may be effective in attenuating insulin resistance and lowering plasma cholesterol levels [17], and with high amylase (Table 1), was used to see if it is the suitable variety for making low GI steam bread. Firstly, steamed wheat breads were made from, purple-grain wheat variety Jizi439, purple-grain variety Chu 20 (micronutriton not riched), the mixture of white grain wheat varieties. They were served with the equivalent of 50 g available carbohydrate of the different breads to ten healthy young volunteers. The postprandial plasma glucose response and the GI value of the products were determined. After chosen the lowest GI value variety, the second experiment was arranged. The effects of different steamed breads on postprandial glucose responses among ten diabetic type 2 subjects were studied by serving three types of foods made from the lowest GI value variety, buckwheat and the mixture of white grain wheat varieties, respectively. The aim was to assess the effect of wheat variety Jizi439 on postprandial plasma glucose in different subjects comparing with buckwheat. In total, twenty participants (10 healthy subjects and 10 type 2 diabetic subjects) were randomly cruited for the study. Ten healthy subjects were normal glucose tolerance, and no overweight and obesity were indicated, there were not diabetes and other historical metabolic diseases.

**Table 1:** Nutritional composition per 100 g of the tested steamed breads

**Nutrition type**	**Wheat variety names**			**Buckwheat**
	**Jizi439**	**Chu20**	**Mixed white wheat**	
Water (g)	49.2	48.5	47.2	48.6
Ash (g)	0.58	0.55	0.61	0.60
protein (g)	10.6	9.8	8.5	6.4
Fat (g)	0.94	0.92	1.01	1.66
Available carbohydrates (g)	37.3	38.9	41.5	39.0
Fibre (g)	1.41	1.38	1.24	3.73
Amylose (g)	18.64	16.83	9.62	-
Resistant starch (g)	1.00	0.85	0.61	0.86
Trivalent organic chromium (ug)	115.2	14.0	-	-
Magnesium (mg)	85.6	82.1	32.9	172.0
Selenium (ug)	5.5	4.8	1.6	3.5
Portion size^a^ steamed bread (g)	134	129	121	128

^a^The portion size of each test was adjusted to contain 50 g available carbohydrates.

## Materials and methods

### Raw materials

Four types of wheat sources, purple-grain wheat variety Jizi439 with riched micronutriton and high amylose (Table 1), purple-grain variety Chu 20 (organic chromium not riched), the mixture (normally high GI) of white grain wheat varieties Shi4185 and Shixin733 (50:50) and buckwheat collected from commercial fields which could effectively reduce diabetes symptom [18], were used in the study. Jizi439 and chu20 were developed by authors [19]. The white wheat varieties were provided by Shijiazhuang Academy of Agriculture and Forestry Sciences. Wheat flour was in a powder form made by removal of 15% bran.

### Test meals

Steamed breads were made from 1kg wheat flour, 500 g water and 6 g dry yeast, The ingredients were mixed and kneaded for 8-10 min, The dough then was fermented for 60 min at 32°C and 75% relative humidity. Then the dough was divided into pieces (50 g available carbohydrate), dough pieces were placed in steamer, the water should be boiling and its cover should not be opened during steaming. Steaming takes 20 minutes. The nutrition contents of one piece of steamed bread for each meal were shown in Table 1.

### Subjects

A total of twenty unrelated individuals were randomly recruited by volunteer. They were divided into two groups: Healthy and diabetic subject group.

In the first test, healthy subjects group consisted of 5 males and 5 females (were not in the menstrual cycle) with ages between 23 and 26 years old were used for selecting the lowest GI vareity. The subjects were randomly recruited from Hebei normal universities (undergraduate and postgraduate) based on the following inclusion criteria: (1) normal glucose tolerance confirmed by a 75-g oral glucose tolerance test (OGTT) according to the 1999 World Health Organization criteria (fasting plasma glucose < 6.1 mmol/l and 2-h plasma glucose < 7.8 mmol/l), (2) no family history of T2DM and (3) the body mass index (BMI) ≤ 28.0 kg/m^2^.

Type 2 diabetic subject group consisted of 10 subjects aged between 45 and 65 years old were used for the second test to prove the effect of consumption of wheat steamed bread made from Jizi439 on postprandial plasma glucose. The subjects were randomly recruited from the Endocrinology Department at Hebei General Hospital. Diabetes was diagnosed in accordance with the 1999 World Health Organization criteria (i.e., fasting plasma glucose ≥7.0 mmol/l and/or 2-h plasma glucose ≥ 11.1 mmol/l). Patients diagnosed with T2DM before 30 years of age, with a body mass index (BMI) > 28 kg/m^2^, or clinical findings consistent with type 1 diabetes or other specific forms of diabetes (e.g., maturity onset diabetes of the young) were excluded from this study. Of these, eight subjects took metformin for lowering glucose, while the others were on lifestyle intervention.

Height, weight, waist circumference, and blood pressure were measured before glycemic response test. BMI was calculated by height and weight. Blood samples were collected to measure fasting plasma glucoseand glycated hemoglobin A1c (HbA1c). For subjects without history of diabetes, a 75-g OGTT test was also conducted to confirm 2-h plasma glucoses were within normal range. The clinical characteristics of the subjects are summarized in Table 2. All participants provided written informed consent, and the study protocol was approved by the Ethics Committee of Hebei General Hospital.

**Table 2:** Subjects clinical characteristics

	**Healthy subjects**	**Patients with T2DM**
N (male/female)	10(5/5)	10(7/3)
Age	25 ± 2	55±10
BMI (kg/m^2^)	23.29 ± 3.28	24.31 ± 3.47
Waist circumference (cm)	80.82 ± 6.45	83.31 ± 5.55
Fasting plasma glucose (mmol/l)	4.88 ± 0.41	7.84 ± 1.15
2-h plasma glucose (mmol/l)	5.85 ± 0.98	-
HbA1c (%)	5.40 ± 0.30	6.60 ± 0.50

**Table 3:** Incremental area under the curve of plasma glucose responses and GI value of test foods

**Variety**	**Incremental area under the curve (iAUC) (0-120 min)**	**Glycemic index (GI) (%)**
Glucose	11.56±1.31^c^	100
Jizi 439	8.56±0.96^a^	75±9^a^
Chu 20	9.59±0.91^b^	84±10^b^
The mixture of white wheat	10.68±1.18^c^	93±11^c^

* Data are presented as mean±SD (n=10). Means with different superscript letters different significantly (repeated measures analysis of variance, post hoc Tukey test, P < 0.05). Glucose was used as the reference food.

### Study design

#### Test of glycemic response of healthy subjects

This test was for choosing the low GI food from three test foods made from Jizi439, chu20 and the mixture of white grain bread wheat, respectively. All the ten Healthy subjects were subjected to fasting 8–10 hours prior to the experiment, and no eating or drinking was allowed. The subjects were told to avoid food rich in DF the entire day before the experiment day, and avoid alcohol, smoking and excessive physical exercise on the day before each test and at the test morning, and otherwise as far as possible to maintain their regular life style throughout the entire study [20-23]. Fasting venous blood samples were collected prior to the breakfast. The subjects were then provided with the test foods, a piece of steam bread made from Jizi439 chu20, and the mixture of white grain bread wheat, respectively, which was equivalent of 50 g available carbohydrates or a 50 g glucose in solution. Each subject was served for 250 ml of drinking water. Thereafter the testing subjects were asked to consume steam breads within 10 min and followed by taking venous blood samples at 15, 30, 45, 60, 120 min after meal. Timing for blood samples started with the first bite of the test meal, the subjects were allowed to drink one bite of water during the test interval. Each subject received four tests: one for reference food and three tests for three different test foods. The time interval between two tests was 3 day. Glucose was used as the reference food.

$$\begin{array}{l} \mathrm{GI}=\left[\left(\mathrm{incremental}\;\mathrm{area}\;\mathrm{under}\;\mathrm{glu}\cos e\;\mathrm{curve}\;\mathrm{for}\;\mathrm{test}\;\mathrm{food}\right)\right)/\\ \;\left(\left(\mathrm{incremental}\;\mathrm{area}\;\mathrm{under}\;\mathrm{glu}\cos e\;\mathrm{curve}\;\mathrm{for}\;\mathrm{equal}\;\mathrm{amounts}\;\mathrm{glu}\cos e\right)\right]\\ \;\times 100 \end{array}$$

#### Plasma glucose determination of diabetics

This test was for choosing the best food for control plasma glucose increment from three test foods made from Jizi439 with riched organic chromium which may be effective in attenuating insulin resistance and lowering plasma cholesterol levels [17], the mixture of white grain bread wheat (normally high GI) and buckwheat which could effectively reduce diabetes symptom [18], respectively. Ten diabetic type2 subjects consumed the equivalent of 50 g available Carbohydrates of different steam bread made from wheat variety Jizi439, the mixture of white bread wheat and buckwheat, respectively, at breakfast. Each subject was served for 250 ml of drinking water at the same the time. The tests were performed 6 days apart and started at the same time in the morning. Blood samples were taken for measuring the plasma glucose of each subject 2 h after breakfast. The test was repeated three days continuously in the morning for each type of bread. The time interval between two tests of different bread was 6 days. The first three days was for Jizi439, and then for, the mixture of white grain wheat, and buckwheat.

### Analytical methods of blood samples

Plasma glucose was measured by the glucose oxidase method with a glucose analyzer (Beckman, USA). All samples were assayed in the same batch.

### Chemical analysis of the test products

Starch is solubilized by boiling the sample in aqueous calcium chloride. Interfering substances are removed by preliminary extraction with aqueous alcohol, and treatment of the calcium chloride extract with a suitable precipitant. Optical activity of the extract is measured with a polarimeter, and starch content is calculated thereform(automatic polarimeter WZZ-2, China). The resistant starch content of the products was determined according to Barry et al. [24]. The amylose starch content of the products was determined according to Scott et al. [25]. Fat is extracted with ethyl ether. Ethyl ether is heated and volatilized, then is condensed above the sample. Solvent continuously drips through the sample to extract the fat. Fat content is measured by weight loss of sample or weight of fat removed (Soxhlet extractor, YG-2, China). Protein content was determined by Kjeldahl analysis (Kjeltec Auto 1030 Analyser, Tecator, Höganäs, Sweden). Cr, Mg, Se in products was determined by atomic absorbent spectrophotometry (atomic fluorescence spectrometer, AFS-2201, China).

### Statistical analysis

The data were analyzed with a general linear model (ANOVA) followed by Tukey's multiple comparison test. Values of p ≤0.05 were considered statistical significance. All the statistical analyses were performed using the SPSS software v19 (SPSS Inc, USA).

## Results

### Postprandial plasma glucose responses of healthy subjects

**Figure 1:**
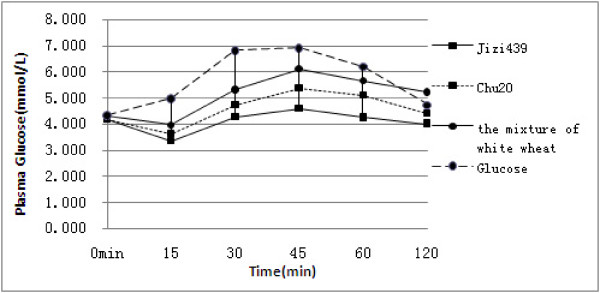
**Postprandial plasma glucose responses of healthy subjects.** Test foods were made from Jizi439, Chu20 and the mixture of white wheat, respectively. Glucose was used as a control. Values are means ±SD, n=10.

Before consuming the different test foods, basal fasting plasma glucose were examined, which were 4.16±0.29, 4.18±0.30, 4.28±0.30, 4.33±0.18 mmol/L for Jizi439, Chu20, the mixture of bread wheat and glucose, respectively. Which were comparable, the postprandial plasma glucose responses curves of healthy subjects for Jizi439, Chu20 and the mixture of white wheat were showed in Figure 1. Generally the plasma glucose level reached peak values in the first 45 min for all the test foods, and decreased slowly thereafter. The plasma glucose response curve showed that glucose resulted the highest among all test foods, followed by the mixture of white wheat, Chu20 and Jizi439. It indicated that the consumption of Jizi439 led to the least increase in plasma glucose at all time points from 15 min to 120 min after the meal. Statistical analysis indicated that the levels of plasma glucose after Jizi439 steamed bread consumption were significantly lower than that of any other testing foods at all the time points (P<0.05).

A mean GI and iAUC values for each steamed wheat bread sample was presented in Table 3. Compared with the reference food (glucose as a reference control), GI and iAUC (15–120 min) values of Jizi439 were significantly lower than that of any food (p<0.05). Although the glucose response for the individual subjects varied to some extent, the lowest GI for Jizi439-based food was observed consistently in all healthy subjects. The GI values of Jizi439 were 19.67% and 10.78% lower (p<0.05) than that of the mixture of white wheat and Chu20, respectively.

### 2. Postprandial plasma glucose responses of diabetic subjects

**Figure 2:**
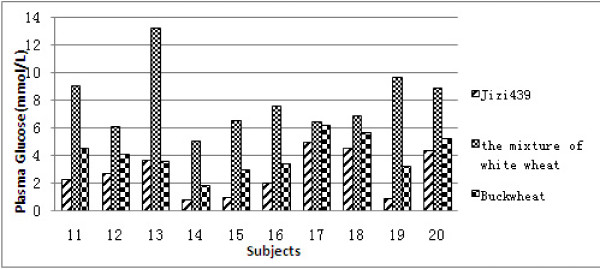
**Postprandial 2 h plasma glucose responses of ten diabetics.** Test foods were made from Jizi439, the mixture of white wheat and buckwheat, respectively.

**Figure 3:**
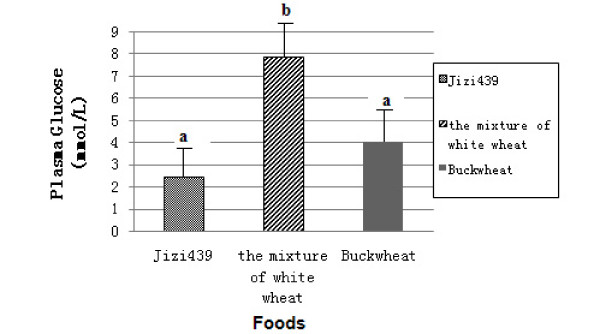
**The comparision of postprandial 2 h plasma glucose of different test foods.** Values are means±SD, n=10. Repeated measures analysis of variance, post hoc Tukey test, P < 0.05, means with different letters different significantly.

A similar test was conducted among ten diabetics. Each diabetic subject consumed test foods (50 g available carbohydrates) made from Jizi439, the mixture of white wheat and buckwheat, respectively. In comparison with the mixture of white wheat, postprandial 2 h plasma ?A3B2 show $132#?>glucose was significantly lower in all diabetic subjects who consumed Ji439 (Figure 2). The average value of postprandial 2 h plasma glucose increment was 2.46, 7.88 and 4.01 mmol/L in different foods (Figure 3). Apparently, consumption of purple-grain bread wheat Jizi439 resulted in the lowest postprandial 2 h plasma glucose, and it was significantly lower than that of the mixture of white wheat. However, no difference was observed between buckwheat and Jizi439 (Figure 3). It is, therefore, concluded that the food made from Jizi439 is an ideal food for diabetics as buckwheat.

## Discussion

GI is important parameter for the evaluation of the “Glycemic potential” of a food. It provides a practical guideline for diabetic people for selecting foods. Foods are categorized into three groups according to their GI [26]. Food with GI < 55 is considered to be low GI food while high GI food is referred to the food with GI >70. Food with GI ranging between 55 and 70 is usually classified as medium GI food. Some studies suggested that whole-grain foods may reduce the glycaemic response and reduce the risk of diabetes [12,13]. That was apparently due to cereal fiber intake. Other studies suggested that the traditional baguette have lower GI in type 2 diabetes subjects than other varieties (classic baguette, loaf of wholemeal bread, loaf of bread fermented with yeast or with leaven, sandwich). These differences might be explained in bread processing and fermentation procedure rather than fibre or resistant starch content [14]. Processing and fermentation procedure of the steamed bread is different from that of the bread. Previous studies have showed that GI of steamed bread made from bread wheat was 98.34, which was classified as high-GI food [15], Yang et al. [27] reported that GI of steamed bread was 88.1±20.2. Thus, white wheat bread is usually defined as a high GI food [20]. In this study, glucose was used as the reference food (only one determination). The GI of steamed bread made from Jizi439 were compared with other varieties, and found that GI of Jizi439 was significantly lower than that of other wheat varieties.

The carbohydrate content of a food and its composition play a major role in influencing the postprandial plasma glucose. Starch is an important carbohydrate source in the human diet. Some studies showed that higher amylose usually results in lower glucose responses [28,29]. Behall et al. reported that high amylose(70% amylose) crackers were significantly lower than the high amylopectin (70% amylopectin) crackers. The present results demonstrated that Jizi439 contains 31.0% amylose (amylose/total starch). Since most wheat cultivars contain 16.18-31.79% amylose in China [30], Jizi439 is considered to be a high amylose wheat variety. This may be the one of the reasons that GI of Jizi439 was lower.

The medical evidence showed that the organic chromium was one of the essential microelements for human body, and played a very important role in maintaining and regulating proper levels of carbohydrate, lipid metabolism and enhancing insulin signaling [26,31,32]. In vitro and in vivo studies suggested that chromium supplements, particularly niacin-bound chromium and/or chromium-nicotinate, may be effective in attenuating insulin resistance and lowering plasma cholesterol levels [17]. In contrast,a few of studies showed that chromium supplementation did not improve glycemic control in type 2 diabetes [33,34]. Nevertheless, most research believed that supplemental Cr is beneficial to diabetes patients [16,17,31,32]. Jizi439 could provide a microelement supplement. This wheat variety offered medicational as well as nutritional values, which made it an ideal food source to prevent diabetes, cardiovascular diseases and enhancing immunity. Therefor, it should be widely used as unique raw material to make various wheat-based end-products, such as bread, steamed bread and noodles.

It was reported that the intake of buckwheat could effectively reduce diabetes symptom [18]. A test that compared the effect of whole buckwheat on plasma glucose with the bread made from refined wheat flour indicated that buckwheat significantly lowered plasma glucose responses [35]; Skrabanja et al. [36] have found that buckwheat may be beneficial to diabetes. In a placebo (sucrose) -controlled study of streptozotocin-diabetic rats, a single dose of buckwheat seed extract lowered plasma glucose levels by 12-19% within 90 and 120 min after administration. In present study, postprandial 2 h plasma glucose level of Jizi439 was lower than that of buckwheat. Therefore, Jizi439 should be also beneficial to diabetes.

## Conclusions

This study indicated that the GI of steamed bread made from Jizi439 was lower than that of Chu20 and mixed white wheat. Jizi439 steamed bread resulted in lower plasma glucose compared with other types of test foods in both healthy and diabetic subjects. So, it is an ideal food source to making steam bread for diabetics.

## Abbreviations

GI: Glycemic index; iAUC: Incremental area under the curve; BMI: Body mass index; OGTT: The oral glucose tolerance test; ANOVA: Analysis of variance.

## Competing interests

The authors declare that they have no competing interests.

## Authors’ contributions

Xingpu Li contributed to the conception and design of this study, led the implementation of the project, and contributed to the modifying of the manuscript, She is the corresponding author. Suque Lan conducted majority of the data collection and analysis and contributed to the writing of the manuscript. Yaning Meng conducted majority of the data collection and analysis and contributed to the writing of the manuscript. Suque Lan and Yaning Meng contributed to the study equally. Yelun Zhang conducted the part of data acquisition and modified the manuscript. Guangyao Song contributed to the modifying of the manuscript, and conducted the part of data acquisition and analysis. Huijuan Ma conducted the part of data acquisition and analysis. All authors has read and approved the final manuscript.
